# “I'm Trying to Reach Out, I'm Trying to Find My People”: A Mixed-Methods Investigation of the Link Between Sensory Differences, Loneliness, and Mental Health in Autistic and Nonautistic Adults

**DOI:** 10.1089/aut.2022.0062

**Published:** 2024-09-16

**Authors:** Lisa Qdtua, Gemma Williams, James Mulcahy, Dennis E.O. Larsson, Marta Silva, Andrew J. Arnold, Hugo D. Critchley, Sarah N. Garfinkel

**Affiliations:** ^1^ Department of Clinical Neuroscience, Brighton and Sussex Medical School (BSMS), University of Sussex, Brighton, United Kingdom.; ^2^ Sussex Partnership NHS Foundation Trust, Brighton, United Kingdom.; ^3^ Faculty of Medicine, Health and Life Science, Swansea University, Swansea, United Kingdom.; ^4^ Department of Neuroscience, Karolinska Institute, Stockholm, Sweden.; ^5^ Institute for Neurosciences, University of Barcelona, Barcelona, Spain.; ^6^ Department of Psychology, University of California San Diego, San Diego, California, USA.; ^7^ Institute of Cognitive Neuroscience, University College London, London, United Kingdom.

**Keywords:** loneliness, autism, anxiety, depression, mixed methods research

## Abstract

**Background:**

Rates of loneliness are substantially higher among autistic compared with nonautistic individuals. This observation refutes the persistent stereotype that autistic individuals are not motivated to seek meaningful social relationships. More plausibly, social environments systematically exclude people with higher levels of sensory differences, impeding on opportunities for autistic individuals to form meaningful relationships. In this study, we sought to quantify the level of distress associated with loneliness (Study A) and provide complementary qualitative insight into experiences of loneliness in relationship to sensory differences in autistic adults (Study B).

**Methods:**

In Study A, *N* = 209 participants completed a range of self-report questionnaires. In Study B, nine autistic adults took part in 10-minute unstructured dyadic conversations around the topic of loneliness. We derived a qualitative understanding of autistic individuals' experience of loneliness, enriched by inductive and deductive analyses.

**Results:**

In Study A, the autistic group showed significantly higher levels of loneliness, loneliness distress, anxiety, depression, and sensory reactivity. We found significant positive correlations between variables, but no group differences in differential relationships. The effect of sensory reactivity on anxiety and depression was mediated by levels of loneliness in both groups. In Study B, autistic participants described the pain of feeling lonely and socially disconnected, while simultaneously experiencing a need for restorative solitude after social overstimulation.

**Discussion:**

Our results indicate that sensory differences are related with higher loneliness and associated poor mental health in both autistic and nonautistic adults. This effect was exacerbated in autistic adults due to higher levels of sensory reactivity. First-hand reports from autistic adults on intense loneliness and the obstructive role of sensory environments refute stereotypes about a lack of social motivation in autistic adults. We conclude that to enable meaningful and inclusive social interaction, a societal effort is needed to create spaces that consider the sensory needs of all neurotypes.

## Community brief

Why is this an important issue?

Research shows that autistic people experience loneliness more often than nonautistic adults. It also shows that sensory differences contribute to higher loneliness, and that both sensory differences and loneliness are related to poor mental health, such as anxiety and depression. However, we do not know if this is unique to autistic adults, or something that also occurs in nonautistic adults. There are also not many studies where autistic people themselves describe loneliness, and how they link their loneliness to sensory differences. Finding out about whether there are differences in autistic and nonautistic people regarding loneliness, and letting autistic people put their thoughts about loneliness into their own words is important to learn more about how we can start to tackle the high rates of loneliness in autistic adults.

What was the purpose of this study?

Our goal was to ask autistic people how they experience loneliness, and to empirically test whether the links between sensory differences, loneliness, and anxiety and depression are present in autistic people only, or also in nonautistic people.

What did the researchers do?

We conducted two separate studies. In Study A, we asked autistic and nonautistic adults to fill out questionnaires about their loneliness, how distressed they are by loneliness, anxiety, depression, and how they experience the sensory environment. In Study B, we asked eight autistic adults to speak with each other and nonautistic participants about their experiences with loneliness. We gave them some conversation starters and then looked for common themes that came up in the conversations.

What were the results of the study?

In Study A, we found that autistic people were more distressed about being lonely, and had higher levels of loneliness, depression, anxiety, and sensory differences than nonautistic participants. However, in both groups, sensory differences were related to higher anxiety and depression, and loneliness was an important influence on this relationship. This finding was supplrted by Study B, where autistic participants described that they often struggle to make meaningful connections because their sensory sensitivities keep them from going out.

What do these findings add to what was already known?

Our findings show in a scientific way that loneliness is just as bad for autistic as nonautistic people. We also find that sensory sensitivities might play a role in making autistic people lonelier.

What are potential weaknesses in the study?

Our study sample does not represent the entire autism spectrum. All our participants were able to speak and travel to the study venues with little support. Our study participants were also mostly White, and our study can, therefore, not speak about the experiences of autistic people across different ethnic groups.

How will these findings help autistic adults now or in the future?

We hope that our findings will support the idea that loneliness is indeed a distressing experience for autistic people, and that society as a whole recognizes the need to create welcoming sensory environments that help to overcome the disconnect that many autistic adults experience.

## Introduction

Loneliness negatively affects physical and mental health, both in neurotypical and neurodivergent individuals.^1,2^ However, rates of loneliness are up to four times higher in autistic than nonautistic individuals,^3^ and autistic individuals have a greater vulnerability to the negative physical and psychological consequences of loneliness.^4^ At the same time, stereotypes persist that autistic people, in contrast to nonautistic individuals, are unmotivated to seek out meaningful social relationships.^5^

In this investigation, we aim to take a dual perspective to address this contradiction. Our first goal is to replicate elevated levels of loneliness in autistic individuals, and then establish quantitatively if there is indeed a difference between autistic and nonautistic people in their loneliness distress and negative health outcomes associated with loneliness. Second, we aim to give first-hand perspectives of autistic people and their experiences of loneliness.

In autistic children and adults, loneliness correlates with increased depression and anxiety,^6–9^ and is associated with an increase in suicidal thoughts and behavior,^10^ and a greater risk of self-harm.^11^ Despite this, a recent systematic review found only a few studies that explicitly investigated loneliness in autistic adults.^2^ This review identified a lack of first-hand descriptions of loneliness in autistic people and a paucity of autism-specific measurement tools. Furthermore, factors linked with increased loneliness in autistic adults included autistic characteristics, heightened anxiety, depression and suicidal ideation, and, importantly to this investigation, sensory avoidance.^2^

Although the relationship between loneliness and poor mental health has come more into focus for the past decade, the stereotype persists that autistic people are disinterested in meaningful social interactions. Indeed, one theory states that a deficit in motivation to engage in the social world is the cause of “impairments” in communication and “disrupted interest” in social engagement.^5^

This theory, called the social motivation deficit hypothesis, is largely at odds with reports from the autistic community of a longing for improved social connection,^12^ and is criticized on this basis.^13^ A discrepancy in mutual understanding between autistic and nonautistic individuals, rather than an autistic deficit in social motivation, offers a more valid framework for appraising communication difficulties and associated feelings of loneliness.^14–17^

The tendency to “other” autistic people,^18^ which likely increases loneliness, is expressed by ascribing social motivation or communication deficits to them, instead of considering the mutual disconnect between neurotypes.^19^ This may cause autistic people to be “abandoned by humanity,” elevating the overrepresentation of loneliness in the autistic population from individual circumstances to “ethical loneliness,” which is how Stauffer describes the rejection of individuals or groups of individuals through repeated societal unethical behavior.^20^ A striking example of this abandonment is the lack of consideration of sensory differences. Many autistic people avoid engaging in social spaces where meaningful interactions could be built as the sensory profile of these places lack provision for neuro-inclusivity.^21,22^

Sensory differences pose a challenge for autistic individuals when seeking meaningful interaction with others. The often-taxing sensory nature of social situations may contribute to increased isolation^23,24^ and thereby exacerbate feelings of depression and anxiety. Sensory differences are classifiable across five hierarchical levels, which include sensory-related neural activity, perceptual reactivity to sensory stimuli, physiological reactivity to sensory input, affective reactivity to sensory input, and behavioral responsivity to sensory input.^25^ Importantly, on all levels, manifestations may be expressed in hyper- (heightened) and/or hypo- (attenuated) sensory differences,^26^ with an individual often experiencing a combination of hyper- and hyporeactivity across sensory levels and modalities.

Such sensory differences are overrepresented in the autistic population^27^ and are associated with a range of anxiety disorders, from specific phobias^28^ to social anxiety.^29^ Increasingly, research identifies sensory reactivity as a predictor of anxiety in autistic people across the lifespan,^30,31^ and the relationship is a recurring theme in qualitative research.^32,33^ This is in line with how autistic adults perceive the chain of causality in linking sensory differences, anxiety, and loneliness. An online survey (*N* = 246) found that a majority of autistic participants perceived sensory hyperreactivity to *cause* anxiety, rather than being *an effect of* anxiety.^34^ Earlier qualitative research supports this further,^24^ and also indicates that behavioral responsivity (sensory seeking or avoiding) is a common strategy of autistic people to alleviate feelings of anxiety.^33,35^

Further research into the potential predictive relationships between poor mental health, loneliness, and sensory reactivity used mediation models to identify intolerance of uncertainty as a significant mediating factor in the relationship between sensory reactivity and anxiety in autistic adults^36^ and in children.^35^ Both hyper- and hypo-sensory reactivity are also linked with increased depressive symptoms in autistic individuals, although this literature focuses on autistic children and young people, and this link is less firmly established in adults.^30,31,37–40^

A recent study found that feelings of loneliness, in young autistic adults, mediated the degree to which sensory avoidance predicted levels of anxiety.^41^ In other words, loneliness explained why feelings of anxiety were increased by the need to avoid distressing sensory experiences, highlighting an important contribution of behavioral responsivity to sensory input to the negative effects of loneliness on mental health.

The concept of loneliness typically refers to the actual or perceived absence of meaningful social connection.^42,43^ However, the size of an individual's social network does not reliably determine satisfaction with social relationships, and being alone (solitude) does not necessarily induce distress at being lonely. In this manner, emotional loneliness (the perceived lack of meaningful social connection) is different from social loneliness (i.e., social isolation).^43^ Although social loneliness often precedes or precipitates emotional loneliness,^44,45^ the latter is found to be associated with increased morbidity and mortality.^1,46^

Therefore, this distinction carries relevant societal and clinical implications for combating emotional loneliness and distress at being lonely. Some psychometric measurement tools reflect this distinction, like the Social and Emotional Loneliness Scale for Adults (SELSA)^47^ or the De Jong Gierveld Loneliness Scale.^48,49^ These instruments are multidimensional, as they measure more than one dimension of loneliness. In contrast, the UCLA Loneliness Scale (UCLA LS)^50^ is unidimensional, focusing on feelings of loneliness in direct relation to the perceived adequacy and feelings about social relationships.^51^

All three of these prevalent loneliness measures touch on the negative affect and distress associated loneliness through items such as “I have an unmet need for a close romantic relationship” (SELSA), “I miss having people around” (De Jong Gierveld Loneliness Scale), or “How often do you feel that your relationships with others are not meaningful?” (UCLA LS). However, we believe that such measures still do not fully capture the important distinction between chosen solitude and distress caused by loneliness. Although potential distress is implied in these questionnaires, there is no explicit measure that assesses whether, and to what degree, loneliness is associated with distress. We, therefore, modified the UCLA LS to include a specific measure of loneliness distress.

Notably, many studies implicating a relationship between sensory differences and loneliness, and the impact on poor mental health, do not compare autistic with nonautistic participants. Combined with the paucity of first-hand descriptions of loneliness from autistic people,^2^ there is a lack of research explicitly showing that, just as the nonautistic population, autistic individuals suffer when there is a lack of meaningful relationships.

In this study, we include a sample of nonautistic adults to test whether the differential relationships between loneliness, distress associated with loneliness, sensory differences, and mental health differ between autistic and nonautistic groups (Study A) and provide complementary authentic descriptions of loneliness from autistic participants (Study B). Case–control studies comparing the experiences of loneliness, associated distress, and the impact on mental health can help to further elucidate whether distress at being lonely is indeed only found in nonautistic adults, or a common association across neurotypes.

Although we did not originally plan Study A and Study B as a joint protocol, we combined them here in a mixed-methods approach to provide a deeper insight into loneliness in autistic adults. Both studies had the shared goal of investigating the social and affective components of loneliness in autistic adults. In Study A, we chose the UCLA LS as a unidimensional measure of loneliness so that we could add an explicit measure of distress at being lonely, instead of relying on the rather implicit measures of distress in existing multidimensional measures.

We modified the UCLA LS together with a Lived Experience Advisory Panel (consisting of four autistic adults) and added the question “How much does this upset you?” after each original item. This enabled us not only to arrive at an explicit measure of loneliness distress, but also to assess quantitatively the stereotype of “chosen solitude without distress” attributable to diminished social motivation in autistic adults.

In Study A, autistic and nonautistic participants completed the modified UCLA LS, in addition to measures of anxiety, depression, and sensory differences. Based on previous research, we hypothesized that autistic participants would display higher scores on all these measures than nonautistic participants. In contrast to the social motivation deficit hypothesis, we expected that loneliness and distress would be highly correlated in both groups, indicating that autistic adults are indeed as distressed by loneliness as nonautistic adults.

Given research indicating a relationship between social avoidance, sensory differences and feelings of loneliness,^41^ we expected to find a positive relationship between these variables in our cohort. We further hypothesized to find a mediating effect of loneliness on the relationship between sensory differences and affective distress (anxiety and depression). In Study B, we undertook a qualitative thematic analysis of transcribed, dyadic conversations held by a group of autistic participants in a different, primary study^17^ with the aim of learning more about how autistic individuals experienced—and made sense of their experiences of—loneliness.

## Methods: Study A

Study A, a quantitative case–control study, was a sub-study of the ADIE (Aligning Dimensions of Interoceptive Experience) clinical trial^52^ and included data collected pre-intervention at the baseline assessment only. In the original ADIE trial, we recruited 121 autistic adults and randomized these participants into two groups, either receiving ADIE or an active control therapy to test effects on anxiety. In addition, we recruited a comparison group of 100 participants without a diagnosis of Autism Spectrum Conditions.

These comparison participants did not take part in the trial but completed the same assessment as ADIE trial participants. As part of the baseline assessment, autistic and comparison participants filled out self-report measures that we partially utilized in this study. We did not include interoceptive measures in this study, which formed part of assessing changes in the ability to accurately perceive bodily signals in the ADIE trial. The original hypotheses, all outcome measures, and procedures of the ADIE trial can be found in a previous publication of results, which includes the trial protocol.^52^ We collected data from both groups between July 2017 and December 2019 at the University of Sussex.

### Transparency and openness

Data for the original ADIE trial is available at https://doi.org/10.25377/sussex.13522259.v1, and data for Study A in this article is available at https://doi.org/10.25377/sussex.20004152. Sample size calculations and preregistered hypotheses (ISRCTN14848787) for the original trial are available in the Supplementary Data of the publication, but we did not conduct separate calculations or preregistrations for this sub-study. The modified UCLA LS questionnaire for Study A can be found in Supplementary Material A in the Supplementary Data.

### Participants

Study A involved 109 adult participants with a formal Diagnostic and Statistical Manual of Mental Disorders/Autism Diagnostic Interview Revised or equivalent confirmed diagnosis of autism (confirmed at screening interview through diagnostic reports from the professional who confirmed the diagnosis provided by participants) and 100 nonautistic controls. All participants were fluent English speakers. We excluded participants if they had a history of past head injury or organic brain disorders, moderate to severe intellectual impairment, epilepsy, or psychotic experiences.

We recruited autistic participants from the Sussex Partnership NHS Foundation Trust (SPFT) Neurodevelopmental Service, advertisements placed on social media, leaflets and posters, local support groups, and through clinicians. We recruited nonautistic participants from staff and students at the University of Sussex and from members of the local community. We matched participants by sex assigned at birth, age, and level of education for the original study. However, only a subset also filled out relevant self-report measures for this study, and we did not match participants for this study.

### Materials and procedure

Study A was approved by the NHS Research Ethics Committee and the Brighton and Sussex Medical School Research Governance and Ethics Committee and was conducted with the sponsorship of SPFT. All participants attended a session at the University of Sussex, or a local SPFT site if autistic participants were unable to travel to University of Sussex. We also gave participants the opportunity to complete self-report measures at home through the online platform Qualtrics to allow for individual preferences.

### Self-report measures

In Study A, all participants provided demographic information about their age, sex assigned at birth, gender identification, and level of education (Table 1). They then completed a series of self-report measures either at their study visit or at home through the online platform Qualtrics if they preferred to fill out questionnaires in their own time. We note that some of the outcome measures used were not developed for or validated in autistic adults and may, therefore, not reflect the characteristics of autistic participants.

**Table 1.** Demographic Characteristics for Study A Participants

Demographic characteristic	Autistic	Nonautistic	χ^2^ (df)	*p*
	*n *(%)	*n *(%)		
Sex assigned at birth			2.32 (1)	0.128
Female	58 (53.2)	64 (64)		
Male	51 (46.8)	36 (36)		
Gender identity			10.60 (2)	0.005
Female	50 (45.9)	64 (64)		
Male	53 (48.6)	36 (36)		
Nonbinary/gender nonconforming	6 (5.5)	—		
Education^a^			18.67 (4)	0.001
GCSE or similar	18 (16.5)	1 (1)		
A-levels or similar	22 (20.2)	30 (30)		
Attended college, no degree	15 (13.8)	8 (8)		
Undergraduate degree	32 (29.4)	37 (37)		
Graduate degree	22 (20.2)	24 (24)		

^a^ Based on UK education system.

#### Spielberger State-Trait Anxiety Inventory

We measured anxiety with the trait anxiety scale of the State-Trait Anxiety Inventory (STAI),^53^ which includes 20 items and is rated on a 4-point scale from “Almost Never” (1) to “Almost Always (4).” Higher scores indicate greater anxiety. This tool was not validated in autistic adults, but we chose it as an outcome measure for the original ADIE trial because it includes components of emotional anxiety and some physiological aspects.^52^ The STAI has good to excellent internal reliability, and acceptable to good test–retest reliability in the general population.^54^

#### Patient Health Questionnaire-9

The PHQ-9 is a 9-item module of the Patient Health Questionnaire for depression and scores nine DSM-IV criteria for major depressive disorder on a scale from 0 to 3 (“Not at all” to “Nearly every day”). The PHQ-9 was validated in a sample of autistic adults, with good psychometric properties.^55^

#### Autism Quotient

The Autism Quotient (AQ) is a 50-item self-administered screening tool for autistic traits.^56^ Responses are scored in a binary manner (0 or 1) from a 4-point item scale ranging from “Definitely agree” to “Definitely disagree.” The AQ, although showing good test–retest reliability and internal consistency, does not always reliably pick up autistic traits, specifically in people assigned female at birth.^57^

#### Glasgow Sensory Questionnaire

The Glasgow Sensory Questionnaire (GSQ) is a 42-item questionnaire that was developed to measure “sensory hyper- and hyposensitivity” in autistic adults across sensory modalities.^58^ However, according to the five-level taxonomy of sensory differences, it would be more accurate to categorize the GSQ as measuring affective reactivity to sensory input.^25^

Total scores, hyper- and hypo sub-scores can be calculated for each modality (visual, auditory, gustatory, olfactory, tactile, vestibular, and proprioceptive), but we only used the total, hyper- and hyporeactivity scores for all modalities combined in this study, as details of specific modalities were out of the scope of this investigation. The GSQ has been translated and validated with good psychometric properties across several languages.^59^

#### UCLA Loneliness Scale

The UCLA LS is one of the most commonly used instruments to measure loneliness but differs from other existing measures in that it is a unidimensional measure of loneliness. It has not been validated in autistic adults,^2^ but shows otherwise excellent psychometric properties.^60^ We added a measure of distress, where each loneliness question from the UCLA LS was followed by a dedicated question to assess distress, that is, “How much does this upset you?” to calculate a parallel “loneliness distress” score (Supplementary Material A in the Supplementary Data). For UCLA LS, two sub-scores (total score of original questions and total score of distress-items) were computed.

### Data analysis

We used SPSS Version 26 for data analysis. We determined group differences in demographic variables using independent samples *t*-tests for continuous measures and chi-square tests for categorical variables. We calculated Cronbach's alpha as an index of internal consistency for all measures in each group. We assessed group differences in loneliness, loneliness distress, affective, and sensory measures using one-way analyses of covariance (ANCOVAs) with added covariates to control for age, gender identity, and level of education. We ran a series of multiple linear regression analyses to test for differential relationships between variables and to identify group differences.

First, we tested whether the relationship between loneliness and loneliness distress was significantly different between groups, by adding loneliness distress as the dependent variable, and loneliness, group, and a loneliness-by-group interaction term as predictors. We then ran separate multiple linear regression models with anxiety and depression as dependent variables, and group, loneliness, and loneliness distress as predictors, respectively. We added a loneliness/loneliness distress-by-group interaction term to assess group differences.

We assumed that autistic traits and sensory differences precede loneliness and loneliness distress, and, therefore, ran separate multiple linear regression models with loneliness/loneliness distress as dependent variables, and group, AQ (autistic traits), total GSQ score, GSQ hyperreactivity, and GSQ hyporeactivity as predictors, respectively, adding an interaction term for all variables. We repeated analyses without the interaction term if it was not statistically significant to establish associations between loneliness, loneliness distress, sensory and affective variables, and autistic traits.

Using SPSS PROCESS,^61^ we conducted a mediation analysis to estimate a potential explanatory effect of loneliness on the relationship between sensory reactivity and affective variables. To identify the most appropriate predictor variable, we first ran separate linear regression analyses with anxiety and depression as dependent variables and GSQ hyperreactivity and GSQ hyporeactivity as predictors. The mediation models then included the sensory variable as predictor, the respective affective variable as outcome, and loneliness as mediator and bootstrapped (*n* = 5000) confidence intervals (CIs).

Because participants in the comparison group were not screened for autism at recruitment, we conducted a sensitivity analysis. We excluded comparison participants who scored above screening cutoff (≥26) on the AQ,^56^ and repeated all analysis steps (Supplementary Material D in the Supplementary Data).

## Results: Study A

We display demographic characteristics and group differences of autistic and nonautistic participants in Table 1. We observed significant differences between groups for age [autistic group age range 18–63, *M* = 33.75, standard deviation (SD) = 12.43; nonautistic group age range 18–64, *M* = 26.72, SD = 10.48; Welch's *t*(206) = −4.43, *p* < 0.001, 95% CI = −10.149 to −3.896], level of education, and gender identity, but not sex assigned at birth.

We display self-report group differences in Table 2. Cronbach's alpha was good to excellent for all measures in both groups. As predicted, autistic traits (measured by AQ) (Supplementary Fig. S1 in the Supplementary Data) were significantly higher in the autistic than the nonautistic group, controlling for age, level of education, and gender identity.

**Table 2.** Group Differences on Self-Report Measures in Study A

Self-report measure	Autistic			Nonautistic			*F *(df)	Mean difference (95% CI)	η_p_^2^
	Mean	SD	α	Mean	SD	α			
AQ	34.94	7.55	0.86	18.35	6.21	0.75	269.74 (1, 204)	16.99 (14.95 to 19.03)	0.57
Trait anxiety	58.39	11.1	0.92	41.55	10.87	0.94	147.72 (1, 204)	19.30 (16.17 to 22.43)	0.42
Depression	12.5	6.17	0.85	5.71	4.35	0.82	75.63 (1, 198)	7.03 (5.44 to 8.63)	0.28
Loneliness	55.9	9.94	0.91	41.62	8.51	0.90	118.05 (1, 200)	15.16 (12.45 to 17.85)	0.38
Loneliness Distress	45.60	13.53	0.95	32.49	8.55	0.90	70.76 (1, 194)	14.42 (11.09 to 17.76)	0.27
GSQ Total	75.06	24.91	0.92	50.33	17.7	0.90	29.44 (1, 87)	28.15 (17.84 to 38.47)	0.25
GSQ Hyper	41.37	14.65	0.90	26.26	11.78	0.90	33.19 (1, 87)	17.31 (11.34 to 23.29)	0.28
GSQ Hypo	33.69	12.1	0.85	24.07	7.62	0.75	17.75 (1, 87)	10.84 (5.73 to 15.96)	0.17

AQ, Autism Quotient; CI, confidence interval; GSQ, Glasgow Sensory Questionnaire; SD, standard deviation.

**FIG. 1.**
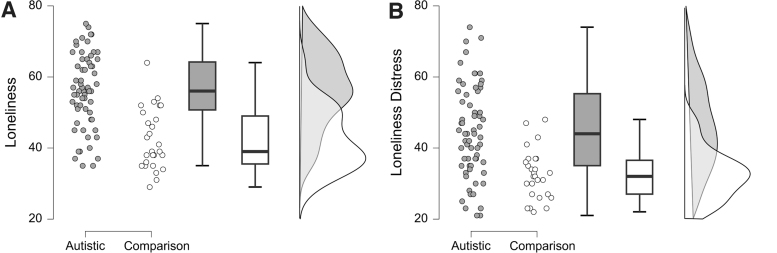
Loneliness **(A)** and loneliness distress **(B)** group differences between autistic and nonautistic participants.

Autistic adults displayed significantly higher trait anxiety (Supplementary Fig. S1 in the Supplementary Data), with 29% having mild, 31% having moderate, and 36% having severe anxiety, compared with 80%, 12%, and 6% in the comparison group, respectively (cutoffs as defined by Emons et al.).^62^ We observed that depression was found to be mild in 5%, moderate in 42%, and severe in 53% of the autistic sample, compared with 41%, 53%, and 6% in the comparison group, respectively (cutoffs as defined by Kroenke et al.^63^) (Supplementary Fig. S1 in the Supplementary Data). Autistic adults also scored higher on loneliness (Fig. 1A), loneliness distress (Fig. 1B), and general sensory reactivity (Supplementary Fig. S1 in the Supplementary Data). All group comparisons controlled for age, gender identity, and level of education (Table 2).

**FIG. 2.**
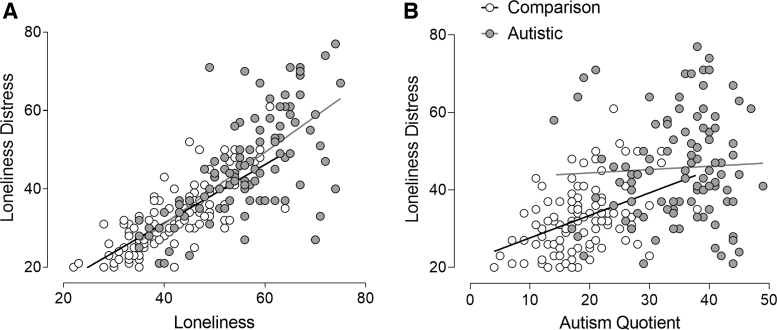
Correlations between loneliness distress and loneliness with no significant group interaction **(A)** and loneliness distress and autism quotient with a significant group interaction **(B)** in autistic and nonautistic participants.

The first regression analysis showed no significant interaction effect of loneliness-by-group, and subsequent analysis identified that although loneliness was a significant predictor of loneliness distress (*β* = 0.756, *p* < 0.001), group (*β* = −0.041, *p* = 0.470) showed no significant effects. The full model explained 61% of the variance [*R*^2^ = 0.61, *F*(3, 198) = 153.86, *p* < 0.001] (Fig. 2A).

Table 3 summarizes results from regression models. We observed no significant interaction effects in models assessing relationships between affective and loneliness variables (all *p* > 0.05). Further analyses without interaction terms indicate that although autistic participants had higher indices on all scores, both groups showed the same differential pattern. Levels of anxiety and depression were significantly associated with loneliness and loneliness distress, and significant group effects show that anxiety and depression are higher in autistic people than the comparison group. Autistic traits, general sensory reactivity, hyperreactivity, and hyporeactivity significantly predicted loneliness in both groups, although loneliness levels were significantly higher in the autistic group.

**Table 3.** Regression Models

Variable	B	95% CI for B		β	*t*	*R^2^*
		LL	UL			
Anxiety						
Model						0.558^***^
Group	8.31	5.08	11.54	0.300^***^	5.07	
Loneliness	0.63	0.49	0.76	0.525^***^	8.88	
Model						0.558^***^
Group	11.04	8.00	14.08	0.394^***^	7.17	
Loneliness Distress	0.50	0.39	0.62	0.466^***^	8.49	
Depression						
Model						0.376^***^
Group	3.90	2.15	5.66	0.308^***^	4.48	
Loneliness	0.20	0.13	0.28	0.375^***^	5.33	
Model						0.437^***^
Group	4.37	2.80	5.93	0.342^***^	5.50	
Loneliness Distress	0.21	0.15	0.27	0.420^**^	6.76	
Loneliness						
Model						0.473^***^
Group	5.28	1.62	8.94	0.227^**^	2.84	
AQ	0.54	0.37	0.71	0.498^***^	6.25	
Model						0.395^***^
Group	10.24	5.61	14.86	0.403^***^	4.40	
GSQ Total	0.153	0.70	0.24	0.388^***^	3.687	
Model						0.380^***^
Group	10.45	5.74	15.16	0.411^***^	4.41	
GSQ Hyper	0.23	0.09	0.37	0.311^***^	3.33	
Model						0.383^***^
Group	11.08	6.55	15.61	0.436^***^	4.86	
GSQ Hypo	0.30	0.13	0.48	0.306^***^	3.42	
Loneliness Distress						
Model						0.611^***^
Group	1.06	−1.83	3.96	0.041	0.73	
Loneliness	0.84	0.72	0.97	0.756^***^	13.42	
Model						0.295^***^
Group	20.88	8.49	33.28	0.800^***^	3.32	
AQ	−0.41	−1.09	0.27	−0.339	−1.19	
Group × AQ	0.50	0.40	0.95	0.392^*^	2.14	
Model						0.204^***^
Group	9.97	4.18	15.75	0.360^***^	3.42	
GSQ Total	0.08	−0.03	0.18	0.157	1.49	
Model						0.206^***^
Group	9.78	3.97	15.59	0.353^***^	3.35	
GSQ Hyper	0.14	−0.03	0.31	0.168	1.60	
Model						0.194^***^
Group	10.74	5.10	16.38	388^***^	3.38	
GSQ Hypo	0.12	−0.10	0.34	0.109	1.07	

^*^ *P*≤0.05; ^**^P≤0.01; ^***^P≤0.001.

LL, lower level of 95% CI; UL, upper level of 95% CI.

We found that the only significant interaction effect in multiple regression models was in the AQ-by-group interaction term predicting loneliness distress (Fig. 2B and Table 3). This finding indicates that loneliness distress is associated with autistic characteristics only in the comparison group, although results could also be explained by a ceiling effect of AQ scores in the autistic group. We did not find significant effects of sensory variables on loneliness distress, although group remained a significant predictor in these models (Table 3).

For both anxiety [*R*^2^ = 0.349, *F*(2, 91) = 23.85, *p* < 0.001] and depression [*R*^2^ = 0.183, *F*(2, 91) = 11.20, *p* < 0.001], sensory hyperreactivity (*β* = 0.506, *p* < 0.001; *β* = 0.333, *p* = 0.25, respectively) was a significant predictor, but not hyporeactivity (*β* = 0.106, *p* = 0.425; *β* = 0.140, *p* = 0.341, respectively). We thus chose sensory hyperreactivity as the predictor variable for the mediation models.

**FIG. 3.**
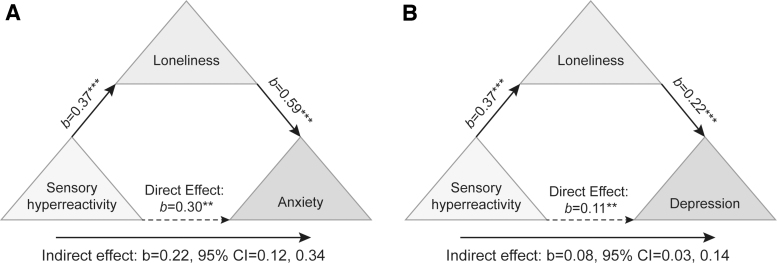
Mediation analyses. Loneliness is a mediator between sensory hyperreactivity and anxiety **(A)** and sensory reactivity and depression **(B)**. Confidence intervals for indirect effect is a bootstrapped confidence interval based on 5000 samples. *P≤0.05; **P≤0.01; ***P≤0.001.

The mediation analyses indicated that the relationships between sensory hyperreactivity, and anxiety and depression were each mediated by loneliness (*b* = 0.221, 95% CI = 0.12 to 0.34; *b* = 0.081, 95% CI = 0.034 to 0.147, respectively). This suggests that higher levels of loneliness may compound the link between heightened sensory hyperreactivity and increased levels of anxiety and depression (Fig. 3).

Sensitivity analyses without comparison participants who scored above screening threshold on the AQ did not change results (Supplementary Material D in the Supplementary Data).

## Discussion: Study A

In Study A, we explored the degree of feelings of loneliness and associated distress in autistic and nonautistic adults. Adding an explicit measure of distress relating to loneliness allowed us to show that autistic adults not only displayed significantly higher levels of loneliness than nonautistic participants, but that their level of loneliness distress was also much greater. This quantification of loneliness distress negates the hypothesis that autistic individuals lack the motivation to seek out meaningful social relationships, refuting the implication that their loneliness is a case of chosen solitude eliciting few feelings of distress.^5^

We did not find a significant group interaction effect on the relationship between loneliness and loneliness distress, demonstrating that loneliness is linked with distress regardless of neurotype. However, due to elevated levels in our autistic participants, higher levels of loneliness were in turn related to higher levels of loneliness distress in this group.

Similarly, we show that there were no group differences in the differential relationships between loneliness, anxiety, depression, and sensory reactivity. As all measures were elevated in the autistic group, only magnitude differences were observed (i.e., greater loneliness associated with greater anxiety, depression, and sensory reactivity in the autistic group) rather than differential relationships between these variables in our two groups. Previous studies focused on within-group designs, showing the impact of sensory reactivity and loneliness on poor mental health in autistic people only. Our findings suggest that similar patterns are found in both autistic and nonautistic people.

The only significant interaction effect we observed was in the relationship between autistic traits and loneliness distress, indicating that even in the absence of an autism diagnosis, loneliness distress monotonically increases with autistic traits. This further strengthens the finding that distress at being lonely is by no means exclusive to nonautistic people, which stands in stark contrast to the social motivation deficit hypothesis. Sensory variables did not predict levels of loneliness distress in either group, indicating that affective, societal, or other aspects are more likely contributors to loneliness distress.

In summary, we show in Study A that while level of loneliness, loneliness distress and associated sensory and affective factors are found to a higher degree in autistic people, the relationship between these variables is not unique to either neurotype. However, these results do not inform about the phenomenology of loneliness in autistic adults. In Study B, we aimed at collating first-hand views from autistic participants on their experiences of loneliness to provide insight into their thought processes and feelings regarding loneliness.

## Methods: Study B

Study B was a sub-study of a doctoral research project in linguistics that investigated differences in communication patterns between autistic and nonautistic speakers.^17^ Autistic and nonautistic participants volunteered to participate in an unstructured conversation for roughly 10 minutes around the topic of loneliness. The original study design was a small-scale linguistic-ethnographic case study using adapted conversational analysis, in which the analysis focused on language use, rather than content of what was said. The analysis reported here in Study B examines the phenomenological content of these conversations at the level of meaning, with the aim of learning how (these) autistic individuals experienced—and made sense of their experiences of—loneliness. For this study, only reports from autistic participants were included.

To make the original study as engaged and participatory as possible within the confines of doctoral research, we set this up as a pilot community engagement project (called “Talking Together”).^17,64^ In a systematic review of interventions for social isolation and loneliness in the elderly, Cattan et al.^65^ found that involving participants in the “planning, developing and delivering of activities” proved the most effective strategy.

The Talking Together project constituted the first stage of these conversations, where individuals could come together to share personal experiences of loneliness: in anticipation of a possible second stage where participants would be supported to co-produce a small-scale community response to loneliness at a local level. As such, we designed conversation prompts (see Materials and Procedure section) to elicit personal experiences of loneliness, more specific thoughts about loneliness in Brighton and Hove (the local city), and to invite ideas around how to address those problems within the city.

However, the conversations rarely addressed local issues, as participants were keener to talk about their personal experienced of loneliness, and in particular how being autistic had a bearing on this. We also observed this tendency in participants' reflections in the communal coffee room directly after conversations and at a follow-up “sense-making” workshop 9 months after the data collection, when participants generally agreed that it had been “a gift” to be able to “share the burden” of their personal loneliness with another person. We include the conversation prompts (Supplementary Material B, pp. 9–10 in the Supplementary Data) here for transparency. Data for Study B were collected in March and April 2019.

### Transparency and openness

We could not make full transcripts from Study B available in accordance with the ethical approval granted by the administering organization, on the basis that even with obviously identifying information reviewed, conversational patterns can be revealing and threaten anonymity. Interview questions for Study B can be found in Supplementary Material B in the Supplementary Data.

### Participants

In Study B, for the purposes of the original linguistic ethnographic study,^17^ we invited eight “core” autistic adult participants (three male and five female) to participate in three short naturalistic conversations, each lasting around 10 minutes, around the topic of loneliness with (1) a familiar and self-chosen conversation partner; (2) an autistic stranger; and (3) a nonautistic stranger.

These conditions were relevant to the primary linguistic study, in which we were interested in whether familiarity and neurotype influenced mutual understanding. We included a ninth autistic participant who attended as the familiar conversation partner of one of the core participants in this thematic analysis. The three conversation conditions were, therefore, incidental to this qualitative study (Study B), and only contributed by providing further time for each participant to discuss and share their ideas.

For the original study, we recruited autistic participants through Assert Brighton and Hove—a local autism support group for autistic adults—and the nonfamiliar nonautistic conversation partners from the University of Brighton. However, for the purposes of this sub-study (Study B), we only included the autistic participants' comments. All participants for both studies provided written informed consent with all procedures.

Owing to the personalized nature of transcript extracts that we included within the article and the need to maintain participant anonymity, there is a limited amount of demographic data that we can share. However, we have included some key demographic information about each participant in Study B in Table 4, with age given as a 10-year window to help maintain anonymity.

**Table 4.** Demographic Notes for Study B Participants

Pseudonym	Demographic notes
Peter	Male, in his 50s, additional learning disability, White.
Miranda	Female, in her mid 30s to mid 40s, White.
Monique	Female, French–English bilingual, in her 50s, White.
Daphne	Female, in her mid 50s to mid 60s, White.
Nigel	Male, in his 50s, White.
Sarah	Female, in her mid 30s to mid 40s, White.
Laura	Female, in her 30s, White.
Molly	Female, in her 20s, White.
Marcus	Male, in his mid 30s to mid 40s, White.

### Materials and procedure

We received ethical approval for Study B by the Tier II Arts and Humanities Ethics Panel at the University of Brighton. Participants attended the premises of the local third sector organization where we recruited participants for Study B in the city centre and held their conversations in a small familiar meeting room. We provided participants with two general prompt questions (Supplementary Material B in the Supplementary Data) for each conversation, that we designed to provide a launching point for conversations. However, once started, conversations tended to veer toward personal experiences of loneliness and social isolation, in particular as an autistic person. The lead researcher did not remain in the room during the conversations.

In total, we digitally recorded and professionally transcribed 245 minutes of naturalistic conversation data. We anonymized participants and allocated pseudonyms. We invited participants to a follow-up “sense-making” workshop several months after the data collection to reflect on their experiences of taking part and to review and contribute to the initial findings and analysis.

### Data analysis

Reflexive thematic analysis is a method for analyzing qualitative data that embraces the researcher's subjectivity as an important *analytic resource* and is compatible with a range of theoretical frameworks.^66,67^ In terms of positionality in Study B, a significant aspect of the lead researcher's subjectivity is that they are themselves autistic. As Bertilsdotter Rosqvist et al.^68^ argue, autism research often occurs within a “neuronormative” academic context, where autism is frequently approached as “an innate deficit in ability and willingness to engage in the social world.”

For the purposes of this study there is an arguable benefit to analyzing the data from the perspective of a researcher who is also autistic themselves.^69^ Underpinned by phenomenology, with the research aim of better understanding how autistic individuals experience and made sense of their loneliness, being able to get “close” to the data^70^ by empathizing with the words and meanings of the participants is essential.

Qualitative reflexive thematic analysis is an iterative and reflexive process involving several stages of development. The first stage of familiarization with the data happened organically, as Study B was a sub-study following the original linguistic analysis, and as such the transcripts were already intimately familiar to the lead researcher (G.W.). During the original linguistic analysis, we logged observations and questions relating to patterns of meaning across the data set in a research journal and revisited this at the start of the thematic analysis.

For the second, coding stage, we initially printed out and annotated transcripts with coloring pencils but then transferred them to the NVivo data analysis programme^71^—software designed to assist in the management of qualitative data sets—and coded them with inductively derived, iteratively developed codes over several passes.

In the early theme development phase, codes seemed to cluster around candidate semantic themes such as “factors contributing to loneliness” (including codes such as “the negative impact of social media,” “mental health issues” and “social isolation” as well as “autism” and “lack of support”); “communicating and connecting” (including codes such as “not being understood”) and a few instances of “possible solutions” to address loneliness (these were on the rare occasion where the conversations flowed toward the localized, community response prompts and included codes such as “a neighbourhood focus,” “low cost” and “time away from social media”).

As we reviewed and reshaped candidate themes, it became clearer that in terms of answering the research question there were two key themes relating to how loneliness was described and experienced that we named “a practical kind of loneliness” and “a deeper yearning for connection.”

Following Study A, we revisited the qualitative data—with its original annotated coding *in situ*—using a deductive approach to search for participant comments that might “support, complement, qualify, or contradict”^70^ patterns identified in Study A. We repeated a similar process to the one outlined earlier, but this time searching the transcripts for comments that specifically spoke to loneliness distress (or a lack thereof), the role of depression and anxiety and feelings around solitude. In a second deductive sweep of the data, we developed one further theme and a related subtheme that we named “seeking solitude” and “overwhelm and the need for solitude.”

## Results and Discussion: Study B

Within the initial inductive analytic phase, we soon decided that two latent codes for loneliness were required, to best reflect two clearly distinct ways that loneliness as a construct was seeming to be described by participants. The first was what we termed a more “practical kind of loneliness”: shaped largely by external factors such as financial, social, and environment barriers to engaging in social activities. The second we named “a deeper yearning for connection” as this included expressions of sorrow and loneliness borne of a desire to connect with others in a meaningful and fulfilling way.

### Theme 1: a practical kind of loneliness

Many of the participants described facing practical barriers to social inclusion, particularly those relating to financial constraints, an absence of affordable and accessible community spaces and a reduced ability to access social spaces in busy noisy urban areas. For example:

Laura^*^: The cost of transport in the city, it's really quite expensive and prohibitive for some people? So, especially if people are out of work or in transient work or zero hour contracts and that kind of thing where they don't know how much how many hours they're gonna get from one month to the next.

Sarah^*^: With the structure of the city in terms of how it's quite.. erm … in order to meet up with people you often have to go out and spend money, unless you go to each other's homes.

Both Laura and Sarah (as well as others) described the challenges associated with being able to afford to access public spaces such as pubs, bars, gigs, or cultural activities that one might share with others such as theater or the cinema. At the time of writing, during a cost-of-living squeeze, such activities may be out of reach for many individuals, but autistic people are especially vulnerable to financial difficulty, with only 22% of autistic people in the United Kingdom in paid work (compared with 81% of nondisabled people: ONS, 2021).^72^

However, financial issues were not the only barriers to accessing public spaces. Daphne, an autistic woman in her early 60s, spoke about the challenges she experiences trying to navigate a busy city centre on her own:

Daphne^*^: Cos I was looking out my living room window and I could see my aunt's house which is the house she used to live in. And I thought that's right over in Hampton Street^*^, and I could see it and I thought well from here I could walk there if it wasn't for all the roads … I could just walk over there …

In Daphne's case she had someone living relatively nearby that she could potentially spend time with if it were not for the fast-moving traffic. Later in the conversation she reminisces about a cold winter “when we had thick, heavy snow” and “it stopped all the traffic.” Not only did it mean that she could suddenly move around the city with ease, but “people seemed to be more friendly” with a “kind of kindness aura” in the city.

Although it may be that strangers were more open to connecting while out enjoying the novelty of the snowfall, it is also possible that the geography of the city *itself* felt less hostile with quieter roads. The built environment is typically designed with a “mythical norm”^22^ in mind, meaning that “everyday living activities such as grocery shopping, buying a coffee, or getting a haircut can become confusing and painful events” for autistic individuals.^21^ For those with sensory and processing differences, something like needing to navigate busy noisy roads alone or manage public transport (as one other participant described) can create insurmountable barriers to engaging in social spaces.

### Theme 2: a deeper yearning for connection

The second and by far the most prevalent type of loneliness that participants described was a deeper yearning for meaningful connection with others and the sense that this was somehow out of reach. When discussing this, participants commonly cited mental health issues such as depression and anxiety as both perceived causes and effects of this kind of deeper loneliness, along with issues directly stemming from being autistic in a predominantly nonautistic world. These specifically autistic challenges included difficulties in connecting with nonautistic people, difficulties in finding others with similar interests, and a lack of being either understood or accepted by wider society.

For example, Monique, a bilingual autistic woman in her early 50s, in discussing her lack of meaningful connections, described the difficulties she experienced in making friends:

… sometimes I have trouble to, erm, to have a conversation or be understood because I don't, mm, have the same thought process? Which makes it weird sometimes and people are wondering “what are you saying?” or “I can't understand what do you mean” or, you know, those kind of things and you have to break it down for people.

It never lasts, or people—once you leave [a job]—they just forget you. Or they say “give me your phone number” and then they never call so I got used to it and I deleted a lot of phone numbers on my phone. It's stupid to pretend you have friends when you haven't got them.

This participant spoke a great deal about not being able to make herself understood, and not because English is not her first language: but because she does not “have the same thought process” as the majority of people she interacts with. In her various conversations she frequently returned to the distress of feeling a “lack of connection” in multiple contexts: with her coworkers, her classmates when she was a child, and even with her older sisters at home when she was small. It was this experiencing of herself as different and unable to make herself understood by those around her that Monique saw as the source of her deep sense of loneliness. This was something that was echoed by many of the participants:

Peter^*^: Cos I feel, you know, say I'm in a group of people and they're all chatting away … cos I'm not on, I'm not on their level […] I feel lonely even though I'm in that, that group: I feel lonely just sitting there.

Sarah^*^: I'm trying to reach out, I'm trying to find my people, but it's not—as you say—it's not connecting deeply within me; it all still feels a bit hopeless and superficial.

Molly^*^: I want to, like, actually like make a connection with someone …

… it's having people to actually talk, like talk openly to.

What is not necessarily obvious from the extracts aforementioned, but implicit (and detailed in other parts of the conversations), is that these interactions are taking place (1) within neurotypical-majority groups and spaces, and (2) within the parameters of a neurotypical-norm dominant society. Autistic people are routinely “othered” in macro and micro social ways, as Sasson et al.^18^ found in one study, which showed that nonautistic people tend to form unconsidered negative opinions about autistic individuals within the first few seconds of meeting them that influence their desire to engage with them.

Moreover, autistic people are more likely than nonautistic people to suffer abuse or interpersonal victimization.^73–78^ Perhaps most importantly, autistic people already exist at a disadvantage in terms of experiencing social connection, due to the fact that the societal norms and values around them are often uncomfortably mismatched with their own.^14^ If you do not have similar “thought processes” to those around you, if you cannot find anyone to share your interests and if the norms and values of the people around you seem alien: it is hard to feel that you belong.

This sense of not-belonging is only compounded when efforts to seek support for the difficult feelings associated loneliness are ignored. Peter, an autistic man in his 50s with additional learning difficulties, shared his confusion and sadness about the lack of support available when he needed it:

… with me having, erm, having, erm, autism, and learning disabilities, I mean I understand a bit more about it today than I did do, but when I wasn't getting the support I felt very lonely … You know, cos, er, you know, you know I didn't have any connection … I was crying out for that support.

… and when you phone it [a helpline] no one ever answers. I mean, I think someone will answer it eventually but from my experience no-one's ever answered it. I've never actually spoken to a person on the other end of the line on this, what-whatever number it was … You know, if people are crying out for help because of how they feel and there's no help then of course they're going to feel lonely or, you know, get into a state …

Peter's experience here echoes the feelings of being abandoned by those in a position to help, and “a loneliness more profound than simple isolation” that Stauffer has termed “ethical loneliness.”^20^

### Theme 3: “seeking solitude” and sub-theme “overwhelm and the need for solitude”

In the second deductive sweep of the data—seeking to find any comments that might “support, complement, qualify, or outright contradict”^70^ patterns identified in the quantitative findings of Study A reported earlier—we developed a further theme, relating to participants' experiences of solitude that we named “seeking solitude.”

What was striking, among conversations rich with descriptions of loneliness and genuine distress about said loneliness, was the frequency with which participants described the pleasure they also took in being alone:

Miranda^*^: I like being on my own.

Monique^*^: I need a lot of time by myself

Nigel^*^: I've spent a lot of time on my own: I'm quite happy on my own a lot of the time

Sarah^*^: I'm very solitary person […] I think my set point is about needing a lot of time for myself

Laura^*^: I'm quite comfortable, erm, with my own company.

This taking pleasure in and seeking time alone did not negate the sometimes extremely distressing experiences of loneliness reported by the autistic participants in their conversations but was nevertheless a common and unifying thread. This need for solitude was only one part of their experiences and participants often mentioned it when trying to communicate what exactly it was about loneliness that was so upsetting. Being alone, they often asserted, was *not* what was distressing: participants often actively enjoyed their own company and wanted to make this clear. Instead, it was something more ephemeral that was painful—loneliness—and this was different to solitude.

Within this theme of seeking solitude, however, we identified some tension. As well as a clear contentment in spending time alone, participants also often described a *need* for solitude resulting from overwhelm: both social and by extension, sensory. To best reflect this, we developed a subtheme we called “Overwhelm and the need for solitude”:

Sarah: Sometimes after spending much time with people … I can feel drained and like I just need some time out.

… so actually most of the friends that I've made are actually autistic, or around somewhere on the autism spectrum, because erm, generally [they're] far more accepting of the fact that sometimes people need space?

Marcus: And er, I think [my loneliness] is to do with my condition though. I just, like, I don't wanna speak to people at work sometimes it's like nooo I don't wanna speak to you just shhhhh […] I mean, I like peace and quiet.

Miranda: … the other night I was in a group of people. It was all women and I struggle a bit with kind of all same sex environments sometimes? And it was a lot of people that I didn't know […] and they were all doing things that I didn't really kind of get and it was all a bit overwhelming. I felt very overwhelmed and I felt very on the edge of it all.

These comments reinforce those we included in the second theme (“A deeper yearning for connection”), that describe the challenges and sense of disconnection often experienced when in neurotypical groups and spaces. Here Sarah, Marcus, and Miranda express a sense of feeling “drained” or “overwhelmed” after a certain point in social interactions: but crucially, when trying to access social connectivity on neurotypical terms. These descriptions, importantly, are means of trying to make sense of their loneliness. The overwhelm that Sarah, Marcus, and Miranda report when attempting to engage in (neurotypical) social environments create significant barriers to connecting with others in fulfilling ways.

Although there are many factors that may contribute to autistic overwhelm, the descriptions aforementioned indicate at least some sensory component. Marcus, for example, paints a portrait of struggling to maintain focus and equilibrium in a busy office environment where people's voices constantly disturb his “peace and quiet.” His self-perceived inability to engage with the clamorous conversations of his colleagues is, he reflects, both related to his being autistic and a likely factor in his social isolation.

Sarah describes feeling drained after spending time in the company of nonautistic others, and observes that it is only other neurodivergent people, in her experience, that can allow space and what might be thought of as time to self-regulate. For anyone who has ever been involved in or witnessed a group of people on an “evening out” in a noisy bar, we can imagine the loud laughter and fast-paced talking surrounding Miranda and how that might be experienced as a sensory experience.

## General Discussion

In this mixed-methods investigation, we aimed to assess whether there were differences in the links between loneliness, associated distress, sensory differences, and mental health between autistic and nonautistic participants, and to provide a complementary qualitative understanding of loneliness in a sample of autistic adults. In Study A, we found that autistic adults showed higher levels of loneliness, associated distress, sensory differences, and poorer mental health compared with nonautistic adults.

Although these levels were elevated in autistic individuals, we observed no group differences in how these variables related to each other, strongly refuting the social motivation deficit hypothesis with its implication that there is a difference in the desire to build and maintain meaningful social connections between autistic and nonautistic groups. In Study B, autistic participants reported yearning for deeper connections, which further contests this stereotype.

Participants in Study B reported a sense of being overwhelmed arising from high-intensity social encounters (i.e., in groups; with strangers; in an office environment). This might represent, in part, an anxiety response and, in part, the experience of receiving “too much information” (to borrow the title of the National Autistic Society's campaign, 2015–2018) or sensory overwhelm. The reported need for solitude (Study B) may thus arise, partially, as a consequence of being overwhelmed during social encounters and a result of the need for low-arousal environments.

We found a similar pattern in Study A, where results of the mediation analysis showed that loneliness is a significant factor in the relationship between sensory reactivity and poor mental health. However, there was no group difference in this relationship, potentially indicating that nonautistic people with high sensory reactivity may have a similar high need for solitude and rest after social encounters.

The exploration of increased loneliness and associated negative consequences in autistic individuals would be incomplete without considering a larger social and societal setting. In the context of “ethical loneliness,”^20^ the fundamental disconnect that many autistic individuals experience stems from an unwillingness of the neurotypical world to make space for, and include, neurodivergent individuals and groups.^18^

Results from both studies show that sensory hyper- and hyporeactivities are important factors associated with loneliness in autistic as well as nonautistic participants. As sensory differences are elevated in autistic participants, they may compound a larger social disconnect,^19^ where experiencing the world differently may lead to a feeling of living in different worlds. This consideration motivates the clinical and societal goal of creating more inclusive environments.

Our studies have several limitations. Despite the relatively large sample size of participants for affective and loneliness measures in Study A, our sample cannot be representative of the entire autistic community for numerous reasons.

In Study A, we excluded individuals with intellectual disabilities that may prevent them from independently reading and answering the survey, and most of our autistic participants used spoken language to communicate and were able to travel independently or with support to study facilities. Our studies, therefore, represent only a specific part of the autistic spectrum. In addition, although none of our nonautistic participants in Study A had an official diagnosis of autism, we did not explicitly screen for autism in the comparison group. Therefore, although on average autistic traits were below the threshold for a suspected diagnosis as measured by AQ, we cannot exclude the possibility that some of them were autistic with absolute certainty.

In Study A, we did not record information on ethnicity or race, making formal inferences on generalizability unfeasible. However, all researchers involved in testing report that most participants in both groups were Caucasian and/or British. Study B involved a very small group of participants, all of whom were White (which may, in part, reflect the wider membership of the local third sector organization where we recruited participants for Study B).

There appear to be continuing barriers to accessing a formal diagnosis of autism and associated support that disproportionately affect people from ethnic minorities. This may be one reason why much autism research inadvertently underrepresents individuals from ethnic minorities, our studies included.^79^ This forms an important limitation on this investigation, and future research must make a concerted effort to include diverse samples of autistic individuals and comparison participants.

Relying exclusively on self-report measures in Study A, associations between variables may have been inflated due to common method variance.^80^ Although the self-report questionnaire we used for measuring sensory reactivity^58^ contains some items touching on behavioral responsivity (seeking/avoidance), it is only validated in adults to provide scores on sensory hyper- and hypo- “sensitivities.” In contrast, the parent-reported GSQ explicitly measures sensory seeking,^81^ a concept that may well play into the relationships we investigated in this article. Future research should thus utilize self-report measures in combination with observational or behavioral tools that record sensory differences at several levels of the hierarchy.^25^

We made our choice of wording for the added questions to the UCLA LS (“How much does this upset you?”) together with our Lived Experience Advisory Panel for the original ADIE trial. At the time, we decided that this sentence would capture feelings of distress toward loneliness. However, our modification of the UCLA LS to incorporate distress requires more formal validation and psychometric evaluation, which is currently underway.^82^

In this psychometric evaluation, we indeed changed the added questions to “How do you feel about this?” with response options ranging from “Very Bad” to “Very Good” instead of “How much does this upset you?” We made this change to reflect broad emotional categories to minimize differences in interpretation and avoid difficulties in identifying feelings, which can be especially prevalent in autistic people.^82,83^

The findings in this article and previous research strongly indicate that loneliness has negative effects on physical and mental health.^1^ Sensory differences and higher indices of anxiety and depression may make autistic individuals particularly susceptible to feeling lonely, yet societal factors underlying loneliness cannot be ignored. Future qualitative and quantitative research should consider an overarching perspective that explicitly scrutinize societal contributions to higher loneliness in the autistic community.

## Conclusion

Taken together, our studies confirm that loneliness is significantly related to feelings of distress and poor mental health in both autistic and nonautistic adults. Moreover, experiencing sensory differences in a world that does not accommodate for variant sensory profiles may drive people to become increasingly isolated, contributing to feelings of loneliness.

As sensory differences are especially prevalent in the autistic community, they may compound other societal, social, and affective factors, ultimately giving rise to higher numbers of loneliness and associated distress. Together with considerations of ethical loneliness and the larger social and societal context, our results highlight the need for welcoming sensory environments to help minimize the disconnect that so many autistic adults experience.

## Notes

Asterisks (^*^) in Study B denote pseudonyms.
