# Supplementary material for: “I'm Trying to Reach Out, I'm Trying to Find My People”: A Mixed-Methods Investigation of the Link Between Sensory Differences, Loneliness, and Mental Health in Autistic and Nonautistic Adults
Source: Autism Adulthood. 2024 Sep 16;6(3):284–99. doi: 10.1089/aut.2022.0062 (PMC11447399; doi:10.1089/aut.2022.0062)

Contents

Supplementary Material A 2
Modified UCLA Loneliness Scale 2
Modified UCLA Loneliness Scale – Scoring sheet 6
Supplementary Material B 11
Prompt questions Study B 11
Supplementary Material C – Figures 12
Supplementary Fig.1 12
Supplementary Material D – Sensitivity Analyses 13
Supplementary Table 1 13
Supplementary Table 2 13
Supplementary Table 3 14
Supplementary Fig. 2 15

Supplementary Material A

Modified UCLA Loneliness Scale

Instructions: The following statements describe how people sometimes feel. For each statement, please indicate how often you feel the way described by one of the options and how much this upsets you.

1. How often do you feel that you are “in tune” with other people around you?

Never	Rarely	Sometimes	Always
◻	◻	◻	◻

1a. How much does this upset you?

Not at all	Somewhat	Quite a lot	Extremely
◻	◻	◻	◻

2. How often do you feel that you lack companionship?

Never	Rarely	Sometimes	Always
◻	◻	◻	◻

2a. How much does this upset you?

Not at all	Somewhat	Quite a lot	Extremely
◻	◻	◻	◻

3. How often do you feel that there is no one you can turn to?

Never	Rarely	Sometimes	Always
◻	◻	◻	◻

3a. How much does this upset you?

Not at all	Somewhat	Quite a lot	Extremely
◻	◻	◻	◻

4. How often do you feel that you feel alone?

Never	Rarely	Sometimes	Always
◻	◻	◻	◻

4a. How much does this upset you?

Not at all	Somewhat	Quite a lot	Extremely
◻	◻	◻	◻

5. How often do you feel part of a group of friends?

Never	Rarely	Sometimes	Always
◻	◻	◻	◻

5a. How much does this upset you?

Not at all	Somewhat	Quite a lot	Extremely
◻	◻	◻	◻

6. How often do you feel that you have a lot in common with the people around you?

Never	Rarely	Sometimes	Always
☐	☐	☐	☐

6a. How much does this upset you?

Not at all	Somewhat	Quite a lot	Extremely
☐	☐	☐	☐

7. How often do you feel that you are no longer close to anyone?

Never	Rarely	Sometimes	Always
☐	☐	☐	☐

7a. How much does this upset you?

Not at all	Somewhat	Quite a lot	Extremely
☐	☐	☐	☐

8. How often do you feel that your interests and ideas are not shared by those around you?

Never	Rarely	Sometimes	Always
☐	☐	☐	☐

8a. How much does this upset you?

Not at all	Somewhat	Quite a lot	Extremely
☐	☐	☐	☐

9. How often do you feel outgoing and friendly?

Never	Rarely	Sometimes	Always
☐	☐	☐	☐

9a. How much does this upset you?

Not at all	Somewhat	Quite a lot	Extremely
☐	☐	☐	☐

10. How often do you feel close to people?

Never	Rarely	Sometimes	Always
☐	☐	☐	☐

10a. How much does this upset you?

Not at all	Somewhat	Quite a lot	Extremely
☐	☐	☐	☐

11. How often do you feel left out?

Never	Rarely	Sometimes	Always
☐	☐	☐	☐

11a. How much does this upset you?

Not at all	Somewhat	Quite a lot	Extremely
☐	☐	☐	☐

12. How often do you feel that your relationships with others are not meaningful?

Never	Rarely	Sometimes	Always
☐	☐	☐	☐

12a. How much does this upset you?

Not at all	Somewhat	Quite a lot	Extremely
☐	☐	☐	☐

13. How often do you feel that no one really knows you well?

Never	Rarely	Sometimes	Always
☐	☐	☐	☐

13a. How much does this upset you?

Not at all	Somewhat	Quite a lot	Extremely
☐	☐	☐	☐

14. How often do you feel isolated from others?

Never	Rarely	Sometimes	Always
☐	☐	☐	☐

14a. How much does this upset you?

Not at all	Somewhat	Quite a lot	Extremely
☐	☐	☐	☐

15. How often do you feel you can find companionship when you want it?

Never	Rarely	Sometimes	Always
☐	☐	☐	☐

15a. How much does this upset you?

Not at all	Somewhat	Quite a lot	Extremely
☐	☐	☐	☐

16. How often do you feel that there are people who really understand you?

Never	Rarely	Sometimes	Always
☐	☐	☐	☐

16a. How much does this upset you?

Not at all	Somewhat	Quite a lot	Extremely
☐	☐	☐	☐

17. How often do you feel shy?

Never	Rarely	Sometimes	Always
☐	☐	☐	☐

17a. How much does this upset you?

Not at all	Somewhat	Quite a lot	Extremely
☐	☐	☐	☐

18. How often do you feel that people are around you but not with you?

Never	Rarely	Sometimes	Always
☐	☐	☐	☐

18a. How much does this upset you?

Not at all	Somewhat	Quite a lot	Extremely
☐	☐	☐	☐

19. How often do you feel that there are people you can talk to?

Never	Rarely	Sometimes	Always
☐	☐	☐	☐

19a. How much does this upset you?

Not at all	Somewhat	Quite a lot	Extremely
☐	☐	☐	☐

20. How often do you feel that there are people you can turn to?

Never	Rarely	Sometimes	Always
☐	☐	☐	☐

20a. How much does this upset you?

Not at all	Somewhat	Quite a lot	Extremely
☐	☐	☐	☐

Modified UCLA Loneliness Scale – Scoring sheet

1. How often do you feel that you are "in tune" with other people around you?

Never	Rarely	Sometimes	Always
4	3	2	1

1a. How much does this upset you?

Not at all	Somewhat	Quite a lot	Extremely
1	2	3	4

2. How often do you feel that you lack companionship?

Never	Rarely	Sometimes	Always
1	2	3	4

2a. How much does this upset you?

Not at all	Somewhat	Quite a lot	Extremely
1	2	3	4

3. How often do you feel that there is no one you can turn to?

Never	Rarely	Sometimes	Always
1	2	3	4

3a. How much does this upset you?

Not at all	Somewhat	Quite a lot	Extremely
1	2	3	4

4. How often do you feel that you feel alone?

Never	Rarely	Sometimes	Always
1	2	3	4

4a. How much does this upset you?

Not at all	Somewhat	Quite a lot	Extremely
1	2	3	4

5. How often do you feel part of a group of friends?

Never	Rarely	Sometimes	Always
4	3	2	1

5a. How much does this upset you?

Not at all	Somewhat	Quite a lot	Extremely
1	2	3	4

6. How often do you feel that you have a lot in common with the people around you?

Never	Rarely	Sometimes	Always
4	3	2	1

6a. How much does this upset you?

Not at all	Somewhat	Quite a lot	Extremely
1	2	3	4

7. How often do you feel that you are no longer close to anyone?

Never	Rarely	Sometimes	Always
1	2	3	4

7a. How much does this upset you?

Not at all	Somewhat	Quite a lot	Extremely
1	2	3	4

8. How often do you feel that your interests and ideas are not shared by those around you?

Never	Rarely	Sometimes	Always
1	2	3	4

8a. How much does this upset you?

Not at all	Somewhat	Quite a lot	Extremely
1	2	3	4

9. How often do you feel outgoing and friendly?

Never	Rarely	Sometimes	Always
4	3	2	1

9a. How much does this upset you?

Not at all	Somewhat	Quite a lot	Extremely
1	2	3	4

10. How often do you feel close to people?

Never	Rarely	Sometimes	Always
4	3	2	1

10a. How much does this upset you?

Not at all	Somewhat	Quite a lot	Extremely
1	2	3	4

**11. How often do you feel left out?**			
Never	Rarely	Sometimes	Always
1	2	3	4
**11a. How much does this upset you?**			
Not at all	Somewhat	Quite a lot	Extremely
1	2	3	4

**12. How often do you feel that your relationships with others are not meaningful?**			
Never	Rarely	Sometimes	Always
1	2	3	4
**12a. How much does this upset you?**			
Not at all	Somewhat	Quite a lot	Extremely
1	2	3	4

**13. How often do you feel that no one really knows you well?**			
Never	Rarely	Sometimes	Always
1	2	3	4
**13a. How much does this upset you?**			
Not at all	Somewhat	Quite a lot	Extremely
1	2	3	4

**14. How often do you feel isolated from others?**			
Never	Rarely	Sometimes	Always
1	2	3	4
**14a. How much does this upset you?**			
Not at all	Somewhat	Quite a lot	Extremely
1	2	3	4

**15. How often do you feel you can find companionship when you want it?**			
Never	Rarely	Sometimes	Always
4	3	2	1
**15a. How much does this upset you?**			
Not at all	Somewhat	Quite a lot	Extremely
4	2	3	4

16. How often do you feel that there are people who really understand you?

Never	Rarely	Sometimes	Always
4	3	2	1

16a. How much does this upset you?

Not at all	Somewhat	Quite a lot	Extremely
1	2	3	4

17. How often do you feel shy?

Never	Rarely	Sometimes	Always
1	2	3	4

17a. How much does this upset you?

Not at all	Somewhat	Quite a lot	Extremely
1	2	3	4

18. How often do you feel that people are around you but not with you?

Never	Rarely	Sometimes	Always
1	2	3	4

18a. How much does this upset you?

Not at all	Somewhat	Quite a lot	Extremely
1	2	3	4

19. How often do you feel that there are people you can talk to?

Never	Rarely	Sometimes	Always
4	3	2	1

19a. How much does this upset you?

Not at all	Somewhat	Quite a lot	Extremely
1	2	3	4

20. How often do you feel that there are people you can turn to?

Never	Rarely	Sometimes	Always
4	3	2	1

20a. How much does this upset you?

Not at all	Somewhat	Quite a lot	Extremely
1	2	3	4

Scoring

UCLA loneliness score = sum of all original items (Questions 1-20)
UCLA loneliness distress score = sum of all added items (Questions 1a-20a)

Availability

The modified UCLA loneliness scale is free to use. Please cite this paper when using the modified scale.

Supplementary Material B

Prompt questions Study B

We're interested in your experiences of loneliness and any ideas you may have about how to address loneliness in the Brighton & Hove area. Some questions to help you:

What does loneliness mean to you?

What is your experience of loneliness in the Brighton and Hove area?

We're interested in your experiences of loneliness and any ideas you may have about how to address loneliness in the Brighton & Hove area. Some questions to help you:

Do the results of Loneliness Experiment (provided) surprise you?

What do you think could be done in the city to address loneliness?

We're interested in your experiences of loneliness and any ideas you may have about how to address loneliness in the Brighton & Hove area. Some questions to help you:

Do you think loneliness is a problem in Brighton and Hove?

What could be done to encourage strangers to talk together more?

Supplementary Material C – Figures

Supplementary Fig.1

Group differences between autistic and comparison participants. Distribution of scores is shown for autistic and comparison participants

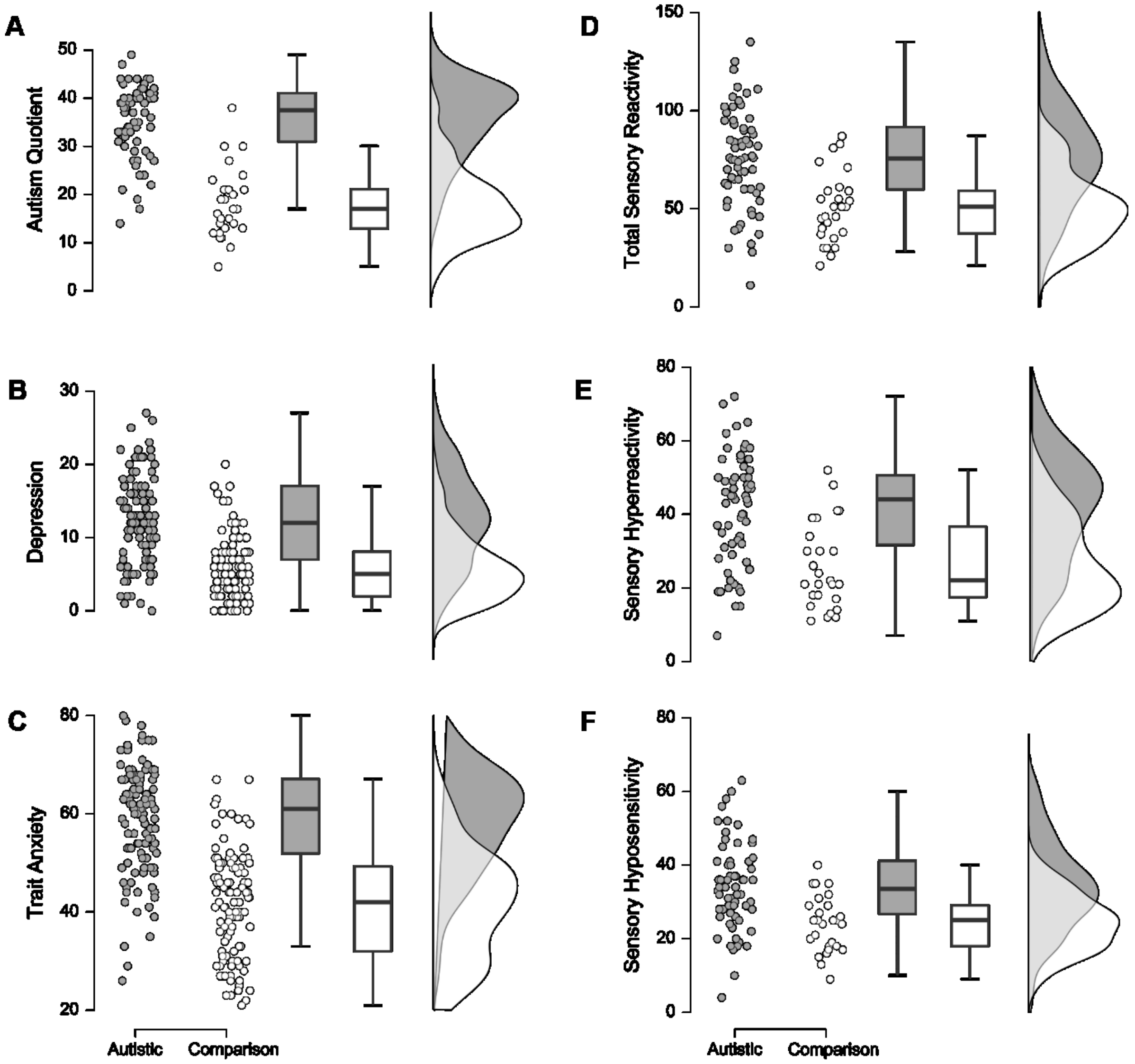

Supplementary Material D – Sensitivity Analyses

We display demographic characteristics and group differences of autistic and non-autistic participants in Supplementary Table 1. We observed significant differences between groups for age (autistic group age range 18-63, M=33.75, SD=12.43; non-autistic group age range 18-63, M=27.13, SD=11.17; *Welch's t*(192)=-3.90, *p*<.001, 95% CI=-10.02, -3.30), level of education, and gender identity, but not sex assigned at birth.

Supplementary Table 1

Demographic group comparisons excluding participants in comparison group who scored above screening threshold on AQ

Demographic characteristic	Autistic	Non-Autistic	χ^2 (df)	*p*
	n (%)	n (%)		
Sex assigned at birth			2.39 (1)	.122
Female	58 (53.2)	55 (65)		
Male	51 (46.8)	30 (35)		
Gender identity			9.79 (2)	.007
Female	50 (45.9)	55 (65)		
Male	53 (48.6)	30 (35)		
Non-binary/gender non-conforming	6 (5.5)	-		
Education[a]			14.64 (4)	.006
GCSE or similar	18 (16.5)	1 (1.2)		
A-levels or similar	22 (20.2)	23 (27.1)		
Attended college, no degree	15 (13.8)	8 (9.4)		
Undergraduate degree	32 (29.4)	32 (37.6)		
Graduate degree	22 (20.2)	21 (24.7)		

Supplementary Table 2

Self-report group comparisons excluding participants in comparison group who scored above screening threshold on AQ

Self-report measure	Autistic			Non-autistic			*F* (df)	*Mean difference (95% CI)*	η_p^2
	Mean	SD	α	Mean	SD	α			
AQ	34.94	7.55	.86	16.53	4.61	.58	365.37 (1, 188)	18.86 (16.92, 20.81)	0.66
Trait anxiety	58.39	11.1	.92	40.45	10.74	.93	158.88 (1, 189)	20.48 (17.27, 23.69)	0.46
Depression	12.5	6.17	.85	5.40	4.16	.82	78.94 (1, 183)	7.45 (5.80, 9.11)	0.30
Loneliness	55.9	9.94	.91	40.59	7.91	.88	134.09 (1, 185)	16.19 (13.43, 18.95)	0.42
Loneliness Distress	45.60	13.53	.95	31.75	8.29	.90	72.89 (1, 179)	15.17 (11.66, 18.67)	0.29
GSQ Total	75.06	24.91	.92	48.65	16.71	.90	28.44 (1, 83)	29.20 (18.31, 40.10)	0.26
GSQ Hyper	41.37	14.65	.90	25.00	11.49	.90	33.45 (1, 83)	18.24 (11.97, 24.51)	0.29
GSQ Hypo	33.69	12.1	.85	23.65	6.67	.75	16.91 (1, 83)	11.07 (5.72, 16.43)	0.17

N=193 (autistic n=109, non-autistic n=85) for AQ, STAI-T (trait anxiety), PHQ-9 (depression), UCLA-L (loneliness), UCLA-D (loneliness distress), GSQ Total (Glasgow Sensory Questionnaire Total score),

GSQ Hyper (Glasgow Sensory Questionnaire Hyperreactivity score), GSQ Hypo (Glasgow Sensory Questionnaire Hyporeactivity score)
N=92 (autistic n=65, non-autistic n=29) for GSQ measures
α= Cronbach's alpha

Supplementary Table 3

Regression analyses excluding participants in comparison group who scored above screening threshold on AQ

Variable	*B*	*95% CI for B*		*β*	*t*	R^2
		LL	*UL*			
Anxiety						
Model						.570***
Group	8.97	5.47	12.47	.317***	5.06	
Loneliness	0.61	0.46	0.76	.511***	8.17	
Model						.577***
Group	12.03	8.83	15.22	.420***	7.96	
Loneliness Distress	0.48	0.36	0.60	.450***	7.96	
Depression						
Model						.387***
Group	4.00	2.01	5.89	.308***	4.12	
Loneliness	0.21	0.13	0.29	.378***	5.06	
Model						.450***
Group	4.26	2.91	6.22	3.51***	5.43	
Loneliness Distress	0.20	0.14	0.27	.419***	6.47	
Loneliness						
Model						.489***
Group	5.81	1.53	10.08	.245*	2.68	
AQ	0.51	0.32	0.70	.484***	5.30	
Model						.397***
Group	11.21	6.30	16.11	.427***	4.54	
GSQ Total	0.14	0.06	0.22	.310***	3.29	
Model						.388***
Group	11.26	6.27	16.24	.429***	4.49	
GSQ Hyper	0.22	0.08	0.36	.295**	3.31	
Model						.382***
Group	12.19	7.39	16.99	.465***	5.05	
GSQ Hypo	0.26	0.08	0.44	.268**	2.92	
Loneliness Distress						
Model						.614***
Group	0.64	-2.54	3.82	.024	0.40	
Loneliness	0.86	0.73	1.00	.768***	12.70	
Model						.308***
Group	23.95	10.16	37.75	.900***	3.43	
AQ	-.62	-1.40	0.17	-.516	-1.55	
Group*AQ	.70	0.10	1.30	.443*	2.31	
Model						.191***
Group	9.81	3.53	16.08	.339**	3.11	
GSQ Total	0.08	-0.03	0.19	.163	1.49	

Model						.196***
Group	9.46	3.15	15.78	.327**	2.98	
GSQ Hyper	0.15	-0.03	0.33	.183	1.66	
Model						.179***
Group	10.78	4.68	16.88	.373***	3.51	
GSQ Hypo	0.11	-0.12	0.34	.104	0.98	

Supplementary Fig. 2

Mediation Analyses excluding participants in comparison group who scored above screening threshold on AQ. Loneliness is a mediator between sensory hyperreactivity and anxiety (A) and sensory hyperreactivity and depression (B).

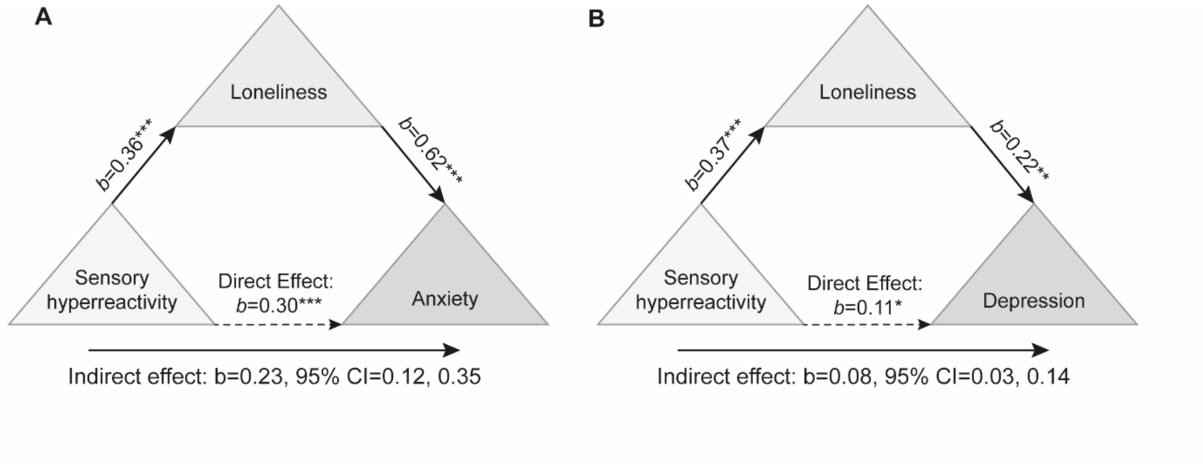

Supplement: Supplementary Data [file aut.2022.0062_suppl_data.pdf]
